# Association Analysis of *WNT3*, *HLA-DRB5* and *IL1R2* Polymorphisms in Chinese Patients With Parkinson’s Disease and Multiple System Atrophy

**DOI:** 10.3389/fgene.2021.765833

**Published:** 2021-11-18

**Authors:** Wei-Ming Su, Xiao-Jing Gu, Yan-Bing Hou, Ling-Yu Zhang, Bei Cao, Ru-Wei Ou, Ying Wu, Xue-Ping Chen, Wei Song, Bi Zhao, Hui-Fang Shang, Yong-Ping Chen

**Affiliations:** ^1^ Department of Neurology, West China Hospital, Sichuan University, Chengdu, China; ^2^ Laboratory of Neurodegenerative Disorders, West China Hospital, Sichuan University, Chengdu, China; ^3^ Rare Disease Center, West China Hospital, Sichuan University, Chengdu, China

**Keywords:** parkinsion’s disease, multiple system atrophy, *Wnt3*, *HLA-DRB5*, *IL1R2*, SNP

## Abstract

**Background:** The association between inflammation and neurodegeneration has long been observed in parkinson’s disease (PD) and multiple system atrophy (MSA). Previous genome-wide association studies (GWAS) and meta-analyses have identified several risk loci in inflammation-associated genes associated with PD.

**Objective:** To investigate whether polymorphisms in some inflammation-associated genes could modulate the risk of developing PD and MSA in a Southwest Chinese population.

**Methods:** A total of 2,706 Chinese subjects comprising 1340 PD, 483 MSA and 883 healthy controls were recruited in the study. Three polymorphisms (rs2074404 GG/GT/TT, rs17425622 CC/CT/TT, rs34043159 CC/CT/TT) in genes linked to inflammation in all the subjects were genotyped by using the Sequenom iPLEX Assay.

**Results:** The allele G of *WNT3* rs2074404 can increase risk on PD (OR: 1.048, 95% CI: 1.182–1.333, *p* = 0.006), exclusively in the LOPD subgroup (OR: 1.166, 95% CI:1.025–1.327, *p* = 0.019), but not in EOPD or MSA. And the recessive model analysis also demonstrated an increased PD risk in GG genotype of this locus (OR = 1.331, *p* = 0.007). However, no significant differences were observed in the genotype distributions and alleles of *HLA-DRB5* rs17425622 and *IL1R2* rs34043159 between the PD patients and controls, between the MSA patients and controls, or between subgroups of PD or MSA and controls.

**Conclusion:** Our results suggested the allele G of *WNT3* rs2074404 have an adverse effect on PD and particularly, on the LOPD subgroup among a Chinese population.

## 1 Introduction

Neurodegenerative diseases such as Parkinson’s disease (PD) and multiple system atrophy (MSA) imposed a severe burden on our aging society. More and more people would suffer from neurodegenerative disorders with increasing life expectancy (Dorsey et al., 2007). Although their causes were not yet fully understood, new genetic insights of PD and some genome-wide association studies (GWAS) have strengthened the evidence that PD has a considerable genetic component (Puschmann, 2013; Chang et al., 2017; Nalls et al., 2019; Blauwendraat et al., 2020). And MSA pedigrees with both autosomal dominant and autosomal recessive inheritance patterns have been reported as well (Wullner et al., 2004; Hara et al., 2007; Itoh et al., 2014).

To date, mechanisms underlying PD and MSA pathogenesis remain not fully understood, but recent studies have shown that neuroinflammation might play an important role in their pathogenesis (Jellinger, 2014; De Virgilio et al., 2016; Gelders et al., 2018). The association between inflammation and neurodegeneration has long been observed in PD (McGeer and McGeer, 2004; Phani et al., 2012; Gelders et al., 2018). In addition, recent studies have shown that inflammation contributions to the abnormal accumulation of α-synuclein, which targets dopamine neurons in the midbrain, forming Lewy bodies in PD (Ohama and Ikuta, 1976; Schain and Kreisl, 2017; Bras et al., 2020). In MSA, which is different from PD, such aggregates usually can be found in oligodendrocytes. However, it has been reported that neuroinflammation also closely links to oligodendroglial α-syncleinopathy (Hoffmann et al., 2019). Therefore, inflammation-related genes may play a part in the pathogenesis of such diseases.

*WNT3* (Wnt Family Member 3) is within a protein-protein interaction network with PD risk genes, such as *LRRK2*, *SNCA* and *MAPT*. It might regulate the gene expression of these PD-related genes in multiple immune cells contributing to PD risk (Witoelar et al., 2017). *HLA-DRB5* (Major Histocompatibility Complex, Class II, DR Beta 5), whose expression products play a central role in the immune system by presenting peptides derived from extracellular proteins, was found to have a possible pathogenic effect Alzheimer’s disease (AD) risk (Karch and Goate, 2015). *IL1R2* (Interleukin 1 Receptor Type 2) is another inflammation-related gene and encodes a cytokine receptor. Polymorphisms, rs2074404 in *WNT3* and rs17425622 in *HLA-DRB5*, have been reported to be associated with PD (Liu et al., 2011; Witoelar et al., 2017). However, such association was not investigated in patients from Chinese. And the previous results of the association between *IL1R2* rs34043159 and PD in the Chinese population were contradictory (Chen et al., 2018; Li et al., 2018; Gao et al., 2020; Hu et al., 2020; Fang et al., 2021).

Hence, in this study, we sought to investigate whether these three polymorphisms in inflammation-associated genes could modulate the risk of developing PD and MSA in a Southwest Chinese population.

## 2 Material and Methods

### 2.1 Subjects

Two independent series of patients, the first cohort of PD diagnosed according to the United Kingdom PD Society Brain Bank Clinical Diagnostic Criteria for PD (Hughes et al., 1992), and the second cohort of MSA meeting the current consensus criteria for the disease (Gilman et al., 2008), were included in this study. Finally, 1823 Chinese subjects comprising 1340 PD, and 483 MSA, were enrolled from the Department of Neurology, West China Hospital of Sichuan University. Detailed clinical variables, such as sex, age, education level, onset age, and initial symptoms, were recorded. In addition, a group of 883 unrelated, age- and sex-matched healthy Chinese controls (HCs) was also recruited for the study as the control group. Neurologists confirmed that all the HCs did not have any neurological disorders. Signed and informed consent forms were obtained from all the subjects. The ethics committee of West China Hospital of Sichuan University approved this study.

### 2.2 Genetic Analysis

As described in our previous studies (Chen et al., 2015; Chen Y. et al., 2020; Chen et al., 2021), peripheral blood leukocytes from subjects were collected for genomic DNA extraction using standard phenol-chloroform procedures. The genotypes for *WNT3* rs2074404, *HLA-DRB5* rs17425622, and *IL1R2* rs34043159 were determined with Sequenom iPLEX Assay technology (Sequenom, San Diego, CA, United States) following the manufacturer’s instructions. Generally, about 10 ng of genomic DNA from each sample was amplified through a locus-specific polymerase chain reaction with primers flanking the targeted sequence designed using Mass ARRAY Assay Design 3.0 software (Sequenom). A 384 element SpectroCHIP bioarray was used to purify and transfer the extension products. Parameters were obtained through matrix-assisted laser desorption/ionization time-of-flight mass spectrometry. Finally, the resultant data were processed and analyzed using Sequenom Mass ARRAY Workstation software. The allele frequencies in five continental populations obtained from 1,000 Genomes and location information of these SNPs was presented in Supplementary Table S1. In consideration of all the three variants located in the non-coding regions of genes, we further predicted where it mapped in the nuclear receptor response elements (NRRE) via NUBIScan15, a computer algorithm to predict DNA recognition sites for nuclear receptors in the regulatory regions of genes, with a threshold of 0.8 raw score by using 100-bp sequence both upstream and downstream of each SNP (Podvinec et al., 2002).

### 2.3 Statistical Analysis

The Hardy–Weinberg equilibrium (HWE) in controls was assessed based on the chi-square test. Age, gender and allele distribution differences between PD patients and controls, and minor allele frequency (MAF) of the SNP were tested by *t*-test or chi-square test when appropriate. As the risk of developing PD and MSA varied for males and females and for subjects of different ages, binary logistic regression analysis was performed to compare the genotype distributions of the SNP between the patients and controls with gender and age as covariates. A two-tailed *p* < 0.05 was considered statistically significant. The statistical power was calculated using PS Power and Sample Size Calculations software (version 3.0.43) (Dupont and Plummer, 1990).

## 3 Results

### 3.1 Clinical Data

A total of 2,706 subjects, including 1,340 PD, 483 MSA, and 883 healthy controls were recruited in this study, and the demographic information is shown in Table 1. The mean age of PD symptoms onset was 56.46 ± 11.47 years, that of MSA was 56.77 ± 8.92 years and that of controls upon recruitment was 56.19 ± 11.05 years. According to previous study (Bonifati et al., 2005), the PD group was divided into early-onset PD (EOPD, onset ≤ 50 years of age) with 379 (28.3%) patients and late-onset PD (LOPD, onset > 50 years of age) subgroup with 961 (71.7%). As for MSA, 254 (52.6%) patients had the MSA-C (MSA with predominant cerebellar features) type and 229 (47.4%) patients had the MSA-P (MSA with predominant parkinsonism) type.

**TABLE 1:** Demographic and clinical characteristics of the patients and controls.

Variables	PD	MSA	Controls
Case (n)	1,340	483	883
Sex, male (%)	698 (52.1%)	242 (50.1%)	433 (49%)
Age at registration (years)	60.25 ± 11.52	59.40 ± 8.64	56.19 ± 11.05
Mean age onset (years)	56.46 ± 11.47	56.77 ± 8.92	—
Mean disease duration (years)	3.80 ± 3.71	2.71 ± 1.94	—
EOPD/LOPD (n)	379/961	—	—
MSA-C/MSA-P (n)	—	254/229	—

PD, parkinson’s disease; MSA, multiple system atrophy; EOPD, early-onset parkinson’s disease; LOPD, late-onset parkinson’s disease; MSA-C, MSA with predominant cerebellar features; MSA-P, MSA with predominant parkinsonism. Data are present as mean ± standard deviation, n or n(%).

### 3.2 Genetic Analysis

The genotype distributions and MAF for the three SNPs in both patients and controls did not deviate significantly from Hardy-Weinberg equilibrium (Table 2). The distributions of *WNT3* rs2074404 GG genotype showed a higher prevalence in the PD patients than the controls (25.67 vs. 20.61%). After adjusted age and gender, further analysis suggested that the allele G of rs2074404 might increase risk on PD (OR: 1.182, 95% CI: 1.048–1.333, *p* = 0.006) and were also significant in the LOPD subgroup (OR: 1.166, 95% CI: 1.025–1.327, *p* = 0.019) (Table 3). In addition, it became highly significant in the recessive model for the PD and LOPD group (OR: 1.331, 95% CI: 1.082–1.637, *p* = 0.007) (Table 4). However, no significant differences were observed in the genotype distributions and alleles of *HLA-DRB5* rs17425622 between the PD patients and controls, between the MSA patients and controls, or between subgroups of PD or MSA and controls (Supplementary Table S2). Likewise, no significant association with PD or MSA was observed for *IL1R2* rs34043159 (Supplementary Table S3). In addition, there were no NRRE of *WNT3*, *HLA-DRB* and *IL1R2* across any sites of the three SNPs via NUBIScan (Supplementary Figure S1).

**TABLE 2:** Distribution of genotype and allele frequencies of rs2074404, rs17425622 and rs34043159 in PD, MSA patients and controls.

SNPs	Genotype			Allele frequencies		H-W x^2^	MAF
rs2074404	GG	GT	TT	G	T
PD	344 (25.67%)	679 (50.67%)	317 (23.66%)	1,367 (51.01%)	1,313 (48.99%)	0.941	0.489
MSA	116 (24.02%)	244 (50.51%)	123 (25.47%)	476 (49.28%)	490 (50.72%)	0.992	0.492
Controls	182 (20.61%)	463 (52.44%)	238 (26.95%)	827 (46.83%)	939 (53.17%)	0.544	0.468
rs17425622	CC	CT	TT	C	T		
PD	15 (1.12%)	249 (18.58%)	1,076 (80.30%)	279 (10.41%)	2,401 (89.59%)	0.999	0.104
MSA	3 (0.62%)	91 (18.84%)	389 (80.54%)	97 (10.04%)	869 (89.96%)	0.743	0.100
Controls	12 (1.36%)	157 (17.78%)	714 (80.86%)	181 (10.25%)	1,585 (89.75%)	0.761	0.103
rs34043159	CC	CT	TT	C	T		
PD	328 (24.48%)	664 (49.55%)	348 (25.97%)	1,320 (49.25%)	1,360 (50.75%)	0.973	0.493
MSA	100 (20.70%)	246 (50.93%)	137 (28.37%)	446 (46.17%)	520 (53.83%)	0.927	0.462
Controls	222 (25.14%)	438 (49.60%)	223 (25.25%)	882 (49.94%)	884 (50.06%)	0.989	0.499

SNPs, single nucleotide polymorphisms; PD, Parkinson’s disease; MSA, multiple system atrophy; H-W, Hardy-Weinberg equilibrium; MAF, minor allele frequency.

**TABLE 3:** Distributions of genotype and allele frequencies of *WNT3* rs2074404 observed in PD, MSA and healthy-matched control.

rs2074404	*n*	Genotype			*p* ^a^	OR (95% CI)	Allele		*p* ^a^	OR (95% CI)
		GG	GT	TT			G	T		
PD	1,340	344	679	317	0.814^b^	1.013 (0.913, 1.123)	1,367	1,313	**0.006** ^b^ ^,^ ^c^	1.182 (1.048, 1.333)
MSA	483	116	244	123	0.848^d^	0.987 (0.864, 1.127)	476	490	0.221^d^	1.103 (0.943, 1.290)
HC	883	182	463	238			827	939		
EOPD	379	93	201	85	0.422^b^	1.067 (0.911, 1.250)	387	371	0.051^b^	1.184 (0.999, 1.404)
LOPD	961	251	478	238	0.309^d^	0.938 (0.829, 1.061)	980	954	**0.019** ^c^ ^,^ ^d^	1.166 (1.025, 1.327)
HC	883	182	463	238			827	939		
MSA-C	254	66	122	66	0.444^b^	0.938 (0.795, 1.106)	254	254	0.207^b^	1.135 (0.932, 1.383)
MSA-P	229	50	122	57	0.642^d^	1.042 (0.875, 1.242)	222	236	0.530^d^	1.068 (0.869, 1.312)
HC	883	182	463	238			827	939		
EOPD	379	93	201	85	0.100	0.796 (0.606, 1.045)	387	371	0.858	1.015 (0.858, 1.201)
LOPD	961	251	478	238			980	954		
MSA-C	254	66	122	66	0.745	1.101 (0.885, 1.369)	254	254	0.635	1.063 (0.826, 1.369)
MSA-P	229	50	57	122			222	236		

a The age and gender were adjusted for as covariates in binary regression models. PD, parkinson’s disease; MSA, multiple system atrophy; EOPD, early-onset parkinson’s disease; LOPD, late-onset parkinson’s disease; MSA-C, MSA with predominant cerebellar features; MSA-P, MSA with predominant parkinsonism; HC, healthy control; OR, odds ratio; CI, confidence interval.

b Comparisons between PD and HC, between EOPD and HC, or between MSA-C and HC.

c Significant (*p* < 0.05).

d Comparisons between MSA and HC, between LOPD and HC, or between MSA-P and HC.

**TABLE 4:** Analysis of genetic models for *WNT3* rs2074404 on risk for PD.

Groups	Model	Genotype	*p* value^a^	OR (95% CI)
PD vs. HC	Dominate	GG + GT vs. TT	0.120	1.170 (0.960, 1.426)
	Recessive	GG vs. TT + GT	**0.007**	1.331 (1.082, 1.637)
EOPD vs. HC	Dominate	GG + GT vs. TT	0.157	1.251 (0.981, 1.705)
	Recessive	GG vs. TT + GT	0.179	1.241 (0.906, 1.698)
LOPD vs. HC	Dominate	GG + GT vs. TT	0.819	1.028 (0.812, 1.302)
	Recessive	GG vs. TT + GT	**0.014**	1.359 (1.065, 1.735)

a The age and gender were adjusted for as covariates in binary regression models. PD, parkinson’s disease; EOPD, early-onset parkinson’s disease; LOPD, late-onset parkinson’s disease; HC, healthy control; OR, odds ratio; CI, confidence interval.

bold value, Significant (*p* < 0.05).

## 4 Discussion

The current study explored the association between polymorphisms in inflammation-related genes and the risk of developing PD and MSA. We replicated the association of *HLA-DRB5* rs17425622 and *IL1R2* rs34043159 with PD, and first analyzed it with MSA. In addition, we firstly studied the possible modification effects of *WNT3* rs2074404 on PD and MSA. The results indicated the *WNT3* rs2074404 could modify the risk for PD, especially for LOPD rather than MSA or EOPD, but not in *HLA-DRB5* rs17425622 and *IL1R2* rs34043159.

*WNT3*, encode secreted signaling protein, that possesses significant functions during neural development, such as neural stem cells (NSC) proliferation, axonal guidance, and neuronal survival (Gonzalez-Fernandez et al., 2014; Duan et al., 2018). A previous study indicated *WNT3* is a candidate risk gene of PD (Liu et al., 2011), and a protein-protein interaction network of *WNT3* with known causative PD genes like *LRRK2* was also perceived (Witoelar et al., 2017). The locus rs2074404 of *WNT3* was previously found to be a CD (Coeliac disease) associated polymorphism (Dubois et al., 2010; Franke et al., 2010; Amundsen et al., 2014). It was also shown that gut inflammation is a main pathological feature occurring in both PD and CD and this inflammation may contribute to α-synuclein aggregation (Dzamko et al., 2017; Drobny et al., 2021). Additionally, some SNPs in genes responsible for binding bacterial metabolites and intestinal homeostasis were associated with PD (Gorecki et al., 2020). All above suggested CD have a link with neurodegenerative diseases, in some respects of genetics and intestinal flora (Voros et al., 2013; Cao et al., 2018; Spielman et al., 2018). Our study found the minor G allele of rs2074404 had a 1.182-bold increased risk of PD, which further supported there may be shared pathways and similar pathogenesis between CD and PD. Analyzing rs2074404 using two genetic models (dominant and recessive) showed that rs2074404 was significantly associated with PD in the recessive mode and with LOPD, suggesting that the G allele was a risk allele for PD.Nevertheless, it didn’t reach significance in the EOPD subgroup. One study indicated that there might be differences in inflammatory responses between EOPD and LOPD, and peripheral immune disorders were more severe in LOPD patients (Jiang et al., 2019). It was also possible that the long-term inflammatory stimulation due to rs2074404 variants increased the risk of LOPD instead of EOPD. Mechanistically, rs2074404, an intron variant mapped in *WNT3*, is not located on the NRRE predicted by NUBIScan, however, we found an NRRE component, ER6 (everted repeats separated by 6 base pairs), is not far upstream from it (Supplementary Figure S1), and the different allele of rs2074404 might affect the ER6 element binding and further influence *WNT3* expression. Furthermore, we found that *WNT3* rs2074404 has a three-dimensional interacting with rs916888 that also has a similar link to *WNT3* rs415430 (Lu et al., 2017) (Supplementary Table S4), which has been reported to be associated with PD in an Ashkenazi Jewish population (Liu et al., 2011). However, whether rs2074404 affects the expression of WNT3, or the fact and potential mechanisms that rs2074404 or rs415430 in *WNT3* were associated with the increased PD risk need more studies. Moreover, this locus makes no difference on MSA onset. Onset age might be one reason, because *WNT3* rs2074404 increased the risk of development for PD, mainly in LOPD, but not in EOPD. For MSA, the mean onset age was younger than that in LOPD, which makes MSA suffer from a relative shorter-term inflammatory stimulation mediated by *WNT3* rs2074404. Even now, the role of neuroinflammation in MSA cannot be ignored. As our previous study has shown, rs3135500 variant in the *NOD2* gene whose products act a role in the immune response might increase the risk for MSA (Cao et al., 2018). However, there were no relevant studies on whether *WNT3* rs2074404 could contribute to the development of MSA. In this study, it was an exploratory study, the smaller sample size of MSA patients might be another reason which contributing the negative results. It is worth point out that our study may be the first one to directly report that rs2074404 is a polymorphism for PD risk with specific data, Therefore, our findings need to be confirmed in more studies with larger sample-size.

**FIGURE 1:**
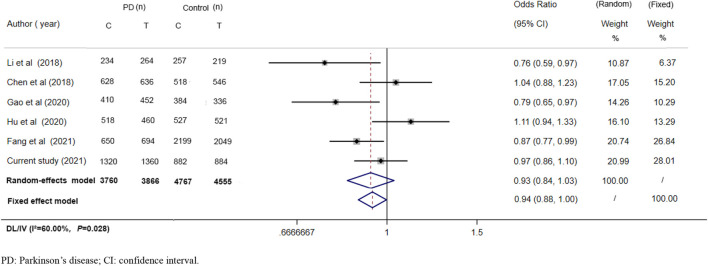
Forest plot for *IL1R2* rs34043159 on PD risk in Chinese. PD: Parkinson’s disease; CI: confidence interval.

*IL1R2* encodes the receptor of IL1 (interleukin-1), a critical inflammation cytokine. The association between IL1 and dopaminergic dysfunction has been reported (Williams-Gray et al., 2016). And this locus has been found to be associated with PD and AD among the Chinese population in some researches (Li et al., 2018; Chen K. et al., 2020; Fang et al., 2021), but in some were not (Chen et al., 2018; Hu et al., 2020). In this current study, rs34043159 was also found to have no effect on both PD and MSA. And then, after a comprehensive searching, we pooled five previous studies including this locus in Chinese and the current analysis (Figure 1; Table 5) together which came out with a nonsignificant result similar to ours, whether performed by random-effects or fixed-effect model. However, using the sample from Chinese (Figure 1, 7,626 allele in patients and 9,322 allele in controls), the statistical power for the rs34043159 polymorphism was 0.677 if the MAF in controls was 38.3% and the OR of developing PD in patients relative to controls 1.08 (Chen et al., 2018; Li et al., 2018; Gao et al., 2020; Hu et al., 2020; Fang et al., 2021), indicated the lack of statistical power to adequately assess the small impact of this risk variant. Further investigations with a larger sample or from experimental evidence are needed to confirm if there is a link between it and PD. In addition, origin-specific effects of the risk locus or potential environmental factors may contribute to these inconsistent results. For MSA, this is the first study to explore rs34043159 on MSA and found out that it is not associated with MSA, whether other loci of *IL1R2* region may make a difference on MSA onset need more evidence.

**TABLE 5:** Characteristics of the included studies for the association between *IL1R2* rs34043159 and PD in Chinese.

Author	Year	Region	PD						Controls					
			CC	CT	TT	Total	C	T	CC	CT	TT	Total	C	T
Li et al.	2018	Southern China	—	—	—	249	234	264	—	—	—	238	257	219
Chen et al.	2018	China	159	310	163	632	628	636	123	272	137	532	518	546
Gao et al.	2020	Eastern China	103	204	124	411	410	452	107	170	83	360	384	336
Hu et al.	2020	China	137	244	108	489	518	460	114	299	111	524	527	521
Fang et al.	2021	Taiwan	177	340	155	672	650	694	478	1,093	553	2,126	2,199	2049
Current study	2021	Western China	328	664	348	1,340	1,320	1,360	222	438	223	883	882	884

PD, parkinson’s disease.

For *HLA-DRB5* rs17425622, it is a risk locus for UC (ulcerative colitis) (Anderson et al., 2011). Braak et al. (2006) suggested that the pathological process in PD originates in the gastrointestinal tract, and then a hypothesis called “gut-brain axis theory” has been proposed, which consists of bidirectional communication between the central nervous system and the enteric nervous system (Carabotti et al., 2015). Indeed, as mentioned above, some researches showed that chronically inflamed gut may trigger α-syn deposition, increase the permeability of the blood-brain barrier leading to neuroinflammation (Lema Tome et al., 2013; Pellegrini et al., 2018). However, whether the SNPs on inflammatory bowel disease genes would increase the risk of PD is worth further exploration. The association of rs17425622 with PD was first explored in a large-scale meta-analysis of genome-wide association, which showed the T allele of rs17425622 could reduce the risk of PD (OR: 0.79, *p* = 8.07*e*−5) in the USA population (Nalls et al., 2014). In addition, a recent study for mapping the HLA locus in PD among Europeans also validated associations for the SNPs rs17425622 (OR: 0.92, 95% CI (0.90, 0.95)) (Yu et al., 2021). However, our preliminary exploration of this locus in the Chinese PD and MSA population did not yield meaningful results. In this study, the statistical power for the rs17425622 polymorphism was 0.857 if the MAF in controls was 21.7% and the OR of developing PD in patients relative to controls 0.79 (Nalls et al., 2014), which indicated the sample size involved in our study was enough. The genetic heterogeneity of different people might be an essential reason for the divergent findings.

The strengths of our study are we included a relatively large and ethnically homogeneous cohort, and *WNT3* rs2074404 related to PD in the Chinese population was tested for the first time. However, this study has some weaknesses or limitations. First, this genetic study did not take the gene-gene or gene-environment interaction into consideration. Second, our study was a single-center study. Thus, more multi-centers and larger studies are expected.

## 5 Conclusion

We found the allele G of *WNT3* rs2074404 was a risk allele for PD and particularly, for the LOPD subgroup. However, we did not detect the significant association of *IL1R2* rs34043159, *HLA-DRB5* rs17425622 with PD and MSA in a Chinese population. More studies for the *WNT3* signaling pathway would be beneficial in developing new therapeutic strategies.

## Data Availability

The original contributions presented in the study are included in the article/Supplementary Material, further inquiries can be directed to the corresponding authors.
