# Supplementary material for: Association Analysis of WNT3, HLA-DRB5 and IL1R2 Polymorphisms in Chinese Patients With Parkinson’s Disease and Multiple System Atrophy
Source: Front Genet. 2021 Nov 18;12:765833. doi: 10.3389/fgene.2021.765833 (PMC8636743; doi:10.3389/fgene.2021.765833)
Supplement: Supplementary file 1 [file DataSheet1.docx]

**Table S1 the information of rs2074404, rs17425622, rs34043159**

| SNPs ID | Gene | location | Position (GRCh38) | Ref/Alt | EAS | AMR | AFR | EUR | SAS |
| --- | --- | --- | --- | --- | --- | --- | --- | --- | --- |
| rs2074404 | *WNT3* | Intron variant | Chr 17:46788073 | T/G | 0.5288 | 0.419 | 0.2534 | 0.2664 | 0.142 |
| rs17425622 | *HLA-DRB5* | NA | chr6:32604184 | T/C | 0.0913 | 0.15 | 0.0318 | 0.1233 | 0.058 |
| rs34043159 | *MAP4K4* | Intron variant | Chr2:101796654 | T/C | 0.4871 | 0.256 | 0.1059 | 0.3539 | 0.508 |

SNPs: single nucleotide polymorphisms; Chr: chromosome; Ref: reference; Alt: alteration; EAS: East Asian; AMR: Admixed American; AFR: African; EUR: South Asian; SAS: South Asian;

the information of these three SNPs from NCBI dsSNP database and allele frequencies in five continental population obtained from 1000 Genomes Phase 3.

**Table S2** **Distributions of genotype and allele frequencies of *HLA-DRB5* rs17425622 observed in PD, MSA and healthy-matched control**

| rs17425622 | n | Genotype | | | *P* | OR (95%CI) | Allele | | *P* | OR (95%CI) |
| --- | --- | --- | --- | --- | --- | --- | --- | --- | --- | --- |
|  |  | CC | CT | TT |  |  | C | T |  |  |
| PD | 1340 | 15 | 249 | 1076 | 0.939 | 1.008(0.826,1.230) | 279 | 2401 | 0.349^#^ | 0.908(0.742,1.111) |
| MSA | 483 | 3 | 91 | 389 | 0.807 | 1.033(0.795,1.344) | 97 | 869 | 0.864^&^ | 0.977(0.754,1.268) |
| HC | 883 | 12 | 157 | 714 |  |  | 181 | 1585 |  |  |
| EOPD | 379 | 3 | 73 | 303 | 0.865 | 0.974(0.718,1.321) | 79 | 679 | 0.896^#^ | 1.019(0.771,1.347) |
| LOPD | 961 | 12 | 176 | 773 | 0.425 | 1.101(0.869,1396) | 200 | 1722 | 0.876^&^ | 1.017(0.822,1.258) |
| HC | 883 | 12 | 157 | 714 |  |  | 181 | 1585 |  |  |
| MSA-C | 254 | 2 | 47 | 205 | 0.853 | 1.031(0.744,1.429) | 51 | 457 | 0.891^#^ | 0.977(0.704,1.356) |
| MSA-P | 229 | 1 | 44 | 184 | 0.767 | 1.054(0.744,1.429) | 46 | 412 | 0.897^&^ | 0.978(0.695,1.375) |
| HC | 883 | 12 | 157 | 714 |  |  | 181 | 1585 |  |  |
| EOPD | 379 | 3 | 73 | 303 | 0.705 | 1.113(0.640,1.933) | 79 | 679 | 0.990 | 1.002(0.761,1.319) |
| LOPD | 961 | 12 | 176 | 773 |  |  | 200 | 1722 |  |  |
| MSA-C | 254 | 2 | 47 | 205 | 0.886 | 0.968(0.625,1.501) | 51 | 457 | 0.998 | 1.000(0.657,1.522) |
| MSA-P | 229 | 1 | 44 | 184 |  |  | 46 | 412 |  |  |

Adjust for age and sex. PD: Parkinson’s disease; MSA: multiple system atrophy; EOPD: early-onset Parkinson’s disease; LOPD: late-onset Parkinson’s disease; MSA-C: MSA with predominant cerebellar features; MSA-P: MSA with predominant parkinsonism; HC: healthy control; OR: odds ratio; CI: confidence interval; ^#^ comparisons between PD and HC, between EOPD and HC, or between MSA-C and HC; ^&^ comparisons between MSA and HC, between LOPD and HC, or between MSA-P and HC;

**Table S3** **Distributions of genotype and allele frequencies of** ***IL1R2* rs34043159 observed in PD, MSA and healthy-matched control**

| rs34043159 | n | Genotype | | | *P* | OR (95%CI) | Allele | | *P* | OR (95%CI) |
| --- | --- | --- | --- | --- | --- | --- | --- | --- | --- | --- |
|  |  | CC | CT | TT |  |  | C | T |  |  |
| PD | 1340 | 328 | 664 | 348 | 0.587 | 1.034(0.916,1.168) | 1320 | 1360 | 0.653^#^ | 0.973(0.863,1.097) |
| MSA | 483 | 100 | 246 | 137 | 0.053 | 1.171(0.998,1.373) | 446 | 520 | 0.059^&^ | 0.860(0.735,1.006) |
| HC | 883 | 222 | 438 | 223 |  |  | 882 | 884 |  |  |
| EOPD | 379 | 89 | 189 | 101 | 0.741 | 1.032(0.856,1.244) | 367 | 391 | 0.482^#^ | 0.941(0.793,1.115) |
| LOPD | 961 | 239 | 475 | 247 | 0.749 | 1.024(0.886,1.183) | 953 | 969 | 0.827^&^ | 0.986(0.866,1.122) |
| HC | 883 | 222 | 438 | 223 |  |  | 882 | 884 |  |  |
| MSA-C | 254 | 53 | 134 | 67 | 0.246 | 1.125(0.922,1.373) | 240 | 268 | 0.284^#^ | 0.898(0.737,1.094) |
| MSA-P | 229 | 47 | 112 | 70 | 0.056 | 1.226(0.995,1.511) | 206 | 252 | 0.058^&^ | 0.819(0.667,1.007) |
| HC | 883 | 222 | 438 | 223 |  |  | 882 | 884 |  |  |
| EOPD | 379 | 89 | 189 | 101 | 0.348 | 0.854(0.614,1.187) | 367 | 391 | 0.586 | 0.954(0.807,1.129) |
| LOPD | 961 | 239 | 475 | 247 |  |  | 953 | 969 |  |  |
| MSA-C | 254 | 53 | 134 | 67 | 0.537 | 1.086(0.836,1.411) | 240 | 268 | 0.481 | 1.095(0.850,1.411) |
| MSA-P | 229 | 47 | 112 | 70 |  |  | 206 | 252 |  |  |

Adjust for age and sex. PD: Parkinson’s disease; MSA: multiple system atrophy; EOPD: early-onset Parkinson’s disease; LOPD: late-onset Parkinson’s disease; MSA-C: MSA with predominant cerebellar features; MSA-P: MSA with predominant parkinsonism; HC: healthy control; OR: odds ratio; CI: confidence interval; ^#^ comparisons between PD and HC, between EOPD and HC, or between MSA-C and HC; ^&^ comparisons between MSA and HC, between LOPD and HC, or between MSA-P and HC;

**Table S4 Genetically association (LD r2 > 0.8) of rs2074404 and rs415430 with rs916888 through 3D chromatin loops.**

| SNPs ID | Population | LD(r2) | Loop type | Loop start | Loop end | Distance | Cell type | Tissue |
| --- | --- | --- | --- | --- | --- | --- | --- | --- |
| rs2074404 | EUR | 0.81719 | Within loop | chr17:44820000-44830000 | chr17:44880000-44890000 | 60000 | H1-hESC | ESC |
| rs415430 | EAS | 1 | Within loop | chr17:44820000-44830000 | chr17:44880000-44890000 | 60000 | H1-hESC | ESC |

SNPs: single nucleotide polymorphism; EUR: European; 3D: three-dimensional; LD: linkage disequilibrium; EAS: East Asian; H1-hESC:H1-human embryonic stem cell; ESC: embryonic stem cell;

**Figure S1** Map of prediction nuclear receptor response elements via NUBIScan


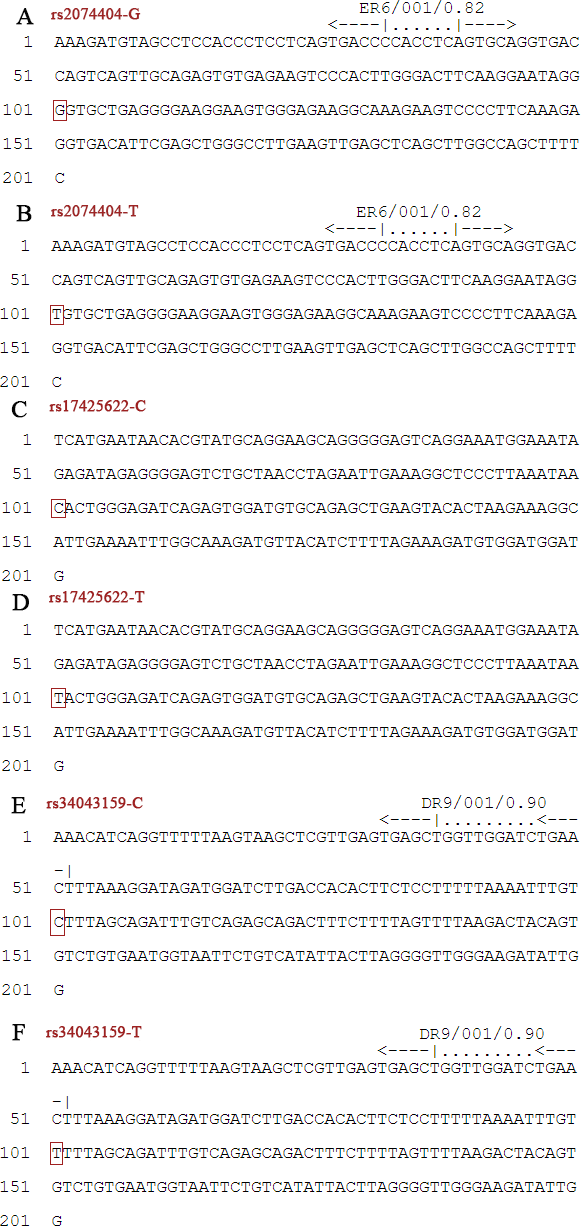


**A, B** Map of prediction nuclear receptor response elements in sequence included rs2074404. **C, D** Map of prediction nuclear receptor response elements in sequence included rs17425622. No element has been found. **E, F** Map of prediction nuclear receptor response elements in sequence included rs34043159. NTOE: The positions of the red boxes are the location of rs2074404, rs17425622 and rs34043159, respectively; ER6: everted repeats separated by 6 base pairs. DR9: direct repeats separated by 9 base pairs.
